# Barriers and framework conditions for the market entry of second-life lithium-ion batteries from electric vehicles

**DOI:** 10.1016/j.heliyon.2024.e37423

**Published:** 2024-09-07

**Authors:** Stefanie Prenner, Florian Part, Sabine Jung-Waclik, Arnaud Bordes, Robert Leonhardt, Aleksander Jandric, Anita Schmidt, Marion Huber-Humer

**Affiliations:** a Brimatech Services GmbH, Lothringerstraße 14/3, 1030, Vienna, Austria; b BOKU University, Institute of Waste Management and Circularity, Muthgasse 107, 1190, Vienna, Austria; c Institut National de l'Environnement Industriel et des Risques (Ineris), Parc Technologique Alata, BP2, 60550, Verneuil-en-Halatte, France; d Bundesanstalt für Materialforschung und -prüfung (BAM), Unter den Eichen 87, 12205, Berlin, Germany

**Keywords:** Electric vehicle, Circular economy, Market entry barrier, Repurposing, Second-life lithium-ion battery, Second-life battery energy storage system

## Abstract

Transition to circular economy for lithium-ion batteries used in electric vehicles requires integrating multiple stages of the value cycle. However, strategies aimed at extending the lifetime of batteries are not yet sufficiently considered within the European battery industry, particularly regarding repurposing. Using second-life lithium-ion batteries (SLBs) before subsequent recycling can offer several advantages, such as the development of sustainable business models, the reduction of emissions, and alignment with UN Sustainable Development Goals 7, 12, and 13. Using expert and problem-centred interviews along with an exploratory workshop, this study guides stakeholders in the battery sector by illustrating the necessary changes for a more holistic circular economy. Moreover, an extended political, economic, social, technological, environmental, legal, and additionally safety-related (PESSTEL) analysis approach is carried out, which has not yet been used in this context. In this process, barriers, as well as necessary institutional framework conditions and organisational requirements for a successful market entry of SLB applications are investigated. Among others, key barriers relate to the competition with first-life applications and safety concerns. SLBs require high manual labour costs for repurposing, along with expenses for expired warranties and re-certifications. Ownership structures in traditional business models often result in SLBs and their corresponding usage data staying under the control of the manufacturers. Market viability, however, requires a level playing field for both first-life and second-life operators as well as circular battery and data-sharing business models. Gathering data on the ageing performance and performing improved safety testing according to test protocols facilitates the reliable assessment of SLBs.

## Highlights

• Repurposing (before recycling) increases sustainability of lithium-ion batteries.
• Ensuring the safety of second-life battery applications is imperative.
• Circular business models are required for second-life applications.
• Original equipment manufacturers frequently act as bottlenecks in the system.
• Promoting modularity and compatibility is crucial for second-life applications.

## 1 Introduction

Electromobility is constantly driving up the production and sale of batteries [1]. With a market share of 60 %, lithium nickel manganese cobalt oxide (NMC) was the predominant battery chemistry used for electric vehicles (EVs) in 2022, followed by lithium iron phosphate (LFP) with a share of around 30 % [2]. Compared to other batteries available on the market, lithium-ion batteries (LIBs) offer a high energy density and a long service life and are therefore currently considered the most promising batteries for EVs [3]. This is also reflected in the increased demand for LIBs in the automotive sector, which has risen by 65 % from around 330 GWh in 2021 to 550 GWh in 2022 [2]. In addition to LIBs, other EV battery types that could be promising in the future, such as redox flow, solid-state, zinc, or sodium-ion, are being investigated at a research and start-up level [2]. In any case, the increasing number of EVs with their associated LIBs will ultimately also lead to an increase in the number of retired end-of-life (EoL) batteries, which currently mainly comprise LIBs [2].

According to the International Energy Agency (IEA), it is estimated that the capacity of retired EV LIBs worldwide will reach 100–120 GWh by 2025 [2]. Usually, this state of retirement, called end-of-first-life (EoFL), is reached by EV LIBs after eight to ten years of operation, depending on the drivers' behaviour and regional conditions [4,5]. This corresponds to a remaining capacity of at least 70 % or a mileage of 160,000 km guaranteed by most European car manufacturers [6]. Up to this point, original equipment manufacturers (OEMs) can guarantee that no ageing knee point is reached that leads to a dramatic drop in capacity and a lower range of an EV. Combining the remaining capacity with the increasing amount of EoFL LIBs available due to the global promotion of EVs, there is a potential for so-called second-life battery (SLB) applications that extend the total battery lifetime [5,[7], [8], [9]]. The extension of the lifetime of EV parts in the automotive industry is addressed specifically by the third principle of the Circular Cars Initiative that stipulates refocusing on higher value retention processes by means of reuse and remanufacturing before recycling [10], and also meets the hierarchical requirements given by the EU Waste Framework Directive [11]. In addition to reuse and remanufacturing, other possible R-strategies to extend the service life of a product include repair, refurbishment, and repurposing [12]. Recycling, as another R-strategy, should only be pursued as a primary waste management strategy when life extension strategies prove technically unfeasible, economically unviable, or are not deemed environmentally favourable [11]. Currently, there are three basic approaches for LIB recycling and metal recovery: pyro- or hydrometallurgical processes, as well as direct recycling [13]. However, it is important to note that this study exclusively focuses on repurposing and does not delve into other R-strategies.

Repurposing implies that cells, modules, packs, or battery systems from EVs that have reached their EoFL are sorted, analysed and reconfigured for another application if the quality is suitable [14]. At the European level, repurposing and ways to facilitate this process are addressed in the amended Battery Regulation [15], for example in Article 45 or 73. Beyond the borders of the EU, the US, for example, has developed a blueprint for LIBs that includes measures to foster second-life concepts [16]. An example of a repurposed SLB application is stationary battery energy storage systems (BESS), which generally require lower energy densities than EV LIBs and are therefore better suited for a second use [4,7,17,18]. This suitability is currently also evident in the market, as the few commercially available SLB applications are mostly BESS. Overall, SLBs and respective applications seem to be promising, especially because of the several benefits they can offer: From an ecological point of view, a life cycle assessment (LCA) case study has shown that repurposing or substituting first-life batteries (FLBs) with SLBs for BESS can reduce the environmental impacts of acidification and global warming potential by 25 % and 16 %, respectively [19]. Economically, the use of SLBs may imply cheaper total EV ownership costs [5,20,21], foster the development of innovative and circular business models [22] and create new ‘green jobs’. In addition, the utilisation of SLBs including recycling can delay and reduce dependence on countries supplying new LIBs [23]. Finally, circular battery markets encompassing FLBs for e-mobility, followed by SLB applications and final recycling, align with the United Nations Development Programme's (UNDP) Sustainable Development Goals (SDGs), particularly ‘Goal 7 Affordable & Clean Energy’, ‘Goal 12 Responsible Consumption and Production’ and ‘Goal 13 Climate Action’. However, a breakthrough in the market has not yet taken place, indicating the existence of barriers to market entry.

The literature, e.g. Refs. [7,8,[24], [25], [26], [27], [28]], has not yet dealt explicitly and in detail with the identification of such market entry barriers as well as institutional framework conditions and necessary organisational requirements as possible solutions to overcome them. In particular, a broad analysis of macroeconomic factors according to an extended political, economic, social, technological, environmental, legal, and additionally safety-related aspects (PESSTEL) approach has not yet been performed. However, there are a few studies that examine to some extent the general barriers related to second-life. In particular: i) Barriers on a generic level as one aspect of the overall picture are highlighted in some reviews on SLBs and their applications [7,8], with one of these reviews offering some possible solutions [7]. ii) Barriers associated with SLB applications, in which a distinction is made between regulatory, economic, environmental, and technological barriers, shedding light on a few advancements required to foster further adoption [24]. iii) Overall challenges for second-life systems, but without analysing the necessary external framework conditions and internal requirements that could help overcome these barriers [28]. iv) Barriers and enablers to second-life applications as a component of a broader research topic [29,30], focusing on the second use of plug-in electric vehicles (PEVs) [29] and exploring the circular economy aspects of LIBs used in mobile and stationary BESS [30]. v) Barriers related to circular business models [[25], [26], [27]], referring to cognitive, organisational, and technical barriers to second use [25], listing a few key barriers in the SLB market [27], and highlighting relevant actors to strengthen the identified drivers and overcome barriers [26]. vi) A reverse logistics implementation barriers analysis on LIBs from EVs, including the R-strategies of reuse, remanufacturing, repurposing, and recycling [31], without specifically addressing market entry barriers and solutions to tackle them. Regarding the methodology employed for data collection, a number of the previously mentioned articles solely rely on literature reviews to examine barriers [7,8,24,27,28]. However, some studies opted for qualitative approaches to gather new data, including interviews [25,30], the Delphi method [26,31], and workshops [25].

Based on the above considerations and findings, the objectives of this study were to: i) identify the perceived and expected market entry barriers for repurposed LIBs from EVs; ii) provide detailed insights into company internal organisational requirements for a large-scale market introduction of SLB applications; and iii) elaborate on the necessary institutional framework conditions for a successful market launch of SLB applications. In this context, institutional aspects refer to the broader political and contextual factors that influence and shape the rules and norms within a given system, while organisational aspects pertain to the internal workings and dynamics of a specific entity operating within that system. To gain first-hand insights and bridge the gaps between research, development, and implementation, an expert and problem-centred interview method, not yet used in this context, was applied. In this respect, the application of an extended PESSTEL approach, including safety-related aspects, represents another novelty of this study. Additionally, an interactive stakeholder workshop was used to validate and supplement findings from the literature and the interviews.

## 2 Material and methods

**Fig. 1:**
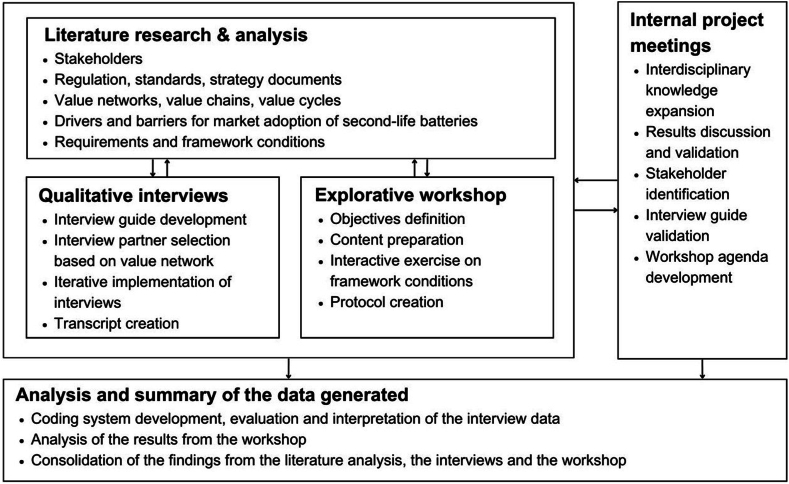
Iterative multi-level research design for data collection and analysis.

**Fig. 2:**
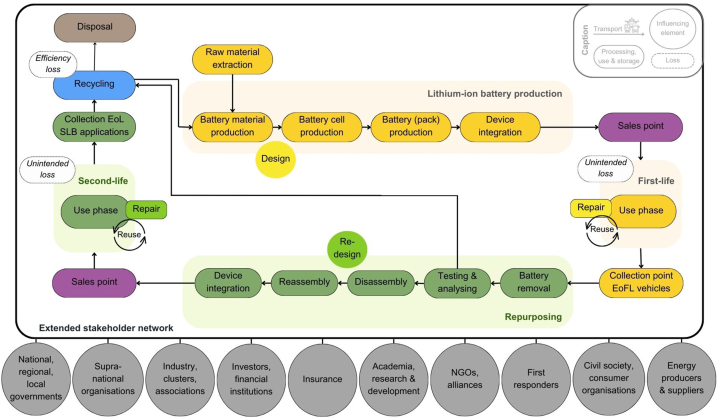
Lithium-ion battery value network with different value cycles and extended stakeholders.

For the study design, a multi-scale research process shown in Fig. 1 was devised, incorporating internal and external feedback loops at various stages. This iterative approach facilitated the discovery of results and ensured optimal engagement with relevant stakeholders. In the first step, literature research and analysis were conducted across a wide range of media and online sources (see Supplementary material). The findings formed the basis for multiple important steps in the study: they provided an overview of the current situation regarding SLB applications and facilitated the development of a value network (see Fig. 2). A ‘value network’ refers to the complex and interconnected system of relationships and interactions among various stakeholders, who collaborate and exchange value to create, produce, and deliver goods or services to the market. It emphasises the interdependence and interrelatedness of all the participants involved in a particular industry or business ecosystem [32].

### 2.1 Qualitative interviews

Interviews, as a method of qualitative research and knowledge acquisition, are ideal for revealing underlying facts and processes that may still be concealed [33]. As a result, qualitative methods facilitate an exploratory approach that generates hypotheses and allows for in-depth exploration of the subject matter [34]. In consideration of the research questions at hand, a methodological approach was adopted, focusing on qualitative expert interviews [35,36] and problem-centred interviews [37].

Based on the value network (see Fig. 2), relevant stakeholder groups were identified which were then used to create a contact database (n = 290). From this database, the interviewees were selected according to the research interest (‘theoretical sampling’), with a particular focus on European stakeholders. The objective was to ensure the heterogeneity of the interviewees and to identify representatives who are typical of their specific interest group. Therefore, actors at different points in the value network were included, so as to document variations that have emerged when adapting to different goals and economic conditions.

An interview guide was developed to ensure a comprehensive and informed approach to gathering insights. Within this interview guide, the situation-problem-implication-need (SPIN) approach, commonly used for selling complex high-technology products, was utilised [38]. Furthermore, this study applied an expanded version of the PESTEL approach [39]. The original PESTEL approach analyses political, economic, social, technological, environmental, and legal influencing factors [39], whereas the adapted approach used in this study additionally includes safety-related factors (i.e., the PESSTEL approach). In the event of conversations hitting roadblocks, the critical incident technique was to be employed, discussing specific cases to reignite the conversation and elicit insights [40]. Due to the iterative character of the interviews, constant feedback loops with the project team in regular online meetings as well as with the literature were possible to further develop the results.

Overall, 13 interviews were conducted anonymously between January and December 2022. A list of the interviewed experts is shown in Table S1 in the Supplementary material. The transcripts generated during the qualitative interviews were analysed using the framework introduced by Mayring (2000) [41]. In this process, the statements of the interviewees were explored, interpreted, categorised, grouped, and summarised. The results in Chapter 3 contain additional literature citations that support the statements of the interviewees.

### 2.2 Explorative workshop

Workshops as a research methodology involve exploring specific scenarios, serving two purposes: maintaining authenticity by meeting participants' expectations and personal interests, as well as generating reliable data for the research objective in the specific domain [42]. In this study, external experts from various sectors such as consultancy, clusters and associations, energy supply, LIB manufacturing, regulation, research and development, standardisation, and repurposing were involved in an exploratory online workshop as part of a corresponding research project. The project team played a crucial role in designing and facilitating this workshop, which was held on October 4, 2022, with 16 participants using the collaborative online tool ‘conceptboard.com’. A special focus of the workshop was on the joint development of institutional framework conditions and internal organisational requirements for overcoming market entry barriers. A recording of the workshop was made to capture the discussions. In addition, an internal project feedback round was conducted to discuss and analyse the protocols and the whiteboard material. The workshop report is available in the technical report by Prenner et al. (2024) [43].

## 3 Results and discussion

Extending an EV LIB's lifetime through repurposing encompasses the removal, testing, and analysis of LIBs to determine their suitability for a second use. After this assessment, the LIBs undergo disassembly and reassembly steps to be finally integrated into an SLB application. Currently, these process steps are not widely adopted as standard practices, making them crucial new roles within a functional circular economy. Fig. 2 shows an idealised LIB value network with several R-strategies and internal value cycles that require a comprehensive stakeholder ecosystem to promote sustainable FLB and SLB solutions.

Given the complex nature of value creation networks and cycles, along with the extensive involvement of multiple stakeholders (see Fig. 2), it is crucial to address various barriers on different levels to ensure the market viability of repurposed LIBs. The qualitative research process has identified perceived and expected market entry barriers, which are explained in the subsequent sub-chapters, following the PESSTEL logic. In addition, each PESSTEL perspective examines institutional framework conditions and organisational requirements to tackle these barriers. It is worth noting that no barriers to market entry were found from an environmental standpoint during the qualitative data collection process.

### 3.1 Political and legal market entry barriers

#### 3.1.1 Insufficient standardisation, regulations, and harmonisation

*“Political framework conditions must be clear or plannable for companies. At present, it is not yet clear in which direction things are really heading.” (*interviewed ExpB; cf. Table S1 in the Supplementary material*)*

Political and regulatory barriers to the market introduction of SLB applications result, according to experts, from Europe's unclear political orientation. This complicates the planning and evaluation of business plans for companies. Furthermore, the current regulatory and standardisation systems for (second-life) LIBs are perceived as immature and regionally inconsistent [44]. This benefits OEMs, who, in the view of experts, are reluctant to be restricted in the design of the batteries and their components. Technology dynamics and varying manufacturer specifications complicate possible standardisation processes of LIBs and their components [45], which, consequently, can hamper the integration of dismantled LIB components into second-life products or systems [44,46]. In addition, without common labels or codes for LIB identification and classification (e.g., LIB architecture, cell chemistry) third parties involved in sorting and repurposing encounter obstacles [23,25]. All these uncertainties in the legal and political framework increase the risk for repurposing companies. Experts stated that under these conditions, an SLB application is ideally already considered in the design phase during the development of the FLB application.

#### 3.1.2 Obtaining and maintaining certifications for second-life batteries

*“There is CE marking for the entire module, which must comply with all DIN and UN standards, including the ADR Directive. As soon as the module is opened, the CE or product status and thus all guarantees are lost.”* (interviewed ExpE; cf. Table S1 in the Supplementary material)

Changing application types necessitates compliance with different regulations and standards (e.g., mobile vs. stationary), potentially requiring additional certifications. Mobile applications generally face more stringent requirements – especially in the automotive sector – than stationary ones, making extra certifications for a repurposed application a potential barrier. However, according to a panel discussion, it should not be particularly difficult to obtain possible additional certifications [47]. In this regard, some companies provide self-declarations stating their compliance with the relevant first-life standards. Moreover, when dismantling the LIB pack down to module or cell level and thus changing the LIB system, certifications (e.g., UN 38.3, the European conformity CE marking) lose their validity, requiring renewal due to safety reasons.

In this context, it is considered beneficial to create the possibility of easier transfer of certifications from FLB to SLB applications. However, due to potential safety risks associated with ageing processes, facilitated transferability of certifications is a controversial issue and should only be possible on the condition that safety is guaranteed.

#### 3.1.3 Transport regulation

*“Replacing cells is not legally possible without further ado, as UN 38.3 is no longer guaranteed. The batteries are changed by a cell replacement and are* thus *no longer considered to have been tested and may therefore not be transported.”* (interviewed ExpF; cf. Table S1 in the Supplementary material)

As far as specific regulations are concerned, the highly regulated dangerous goods transport sector is perceived as a barrier to the market entry of SLB, but also FLB applications [25]. At the United Nations (UN) level, there are overarching Recommendations on the Transport of Dangerous Goods including Model Regulations that all batteries must comply with [48,49]. This is particularly important because no LIBs can be placed on the market if transport is not possible. Furthermore, these recommendations include a Manual of Tests and Criteria [50], of which the series of tests defined in § 38.3 ‘Lithium metal and lithium ion batteries’ have been identified as potential market entry barriers for SLB applications in the interviews and the literature [51]. Although SLBs and repurposing are not explicitly mentioned within these recommendations, the regulation applies to a second use [50]. According to the test series, for example, there is a need to re-evaluate LIBs that correspond to a new ‘type’ as defined in § 38.3.2.2 [50], which means there are changes in the parameters that affect the safety of the battery. The use of second-life components can induce such changes and subsequently require retesting, resulting in additional expenses. Furthermore, determining a battery type for SLBs that is representative of the batteries being transported and tested is complicated by the inhomogeneity of the cells used. Therefore, a presentation by the UN Sub-Committee of Experts on the Transport of Dangerous Goods concluded that the UN Model Regulations might need to be adapted to clarify the testing requirements for LIBs consisting of repurposed cells [52].

#### 3.1.4 New EU battery regulation

*“Recycling has a completely new position, especially* due to *the mandatory recycled content [in the new Battery Regulation]. If second-life quotas were introduced, that would also be a driver that could make a difference.”* (interviewed ExpK; cf. Table S1 in the Supplementary material)

The overarching objective of the European Commission's new Battery Regulation [15] is to ensure that batteries placed on the European market are sustainable and safe throughout their life cycle. Minimum requirements are set for SLBs and corresponding applications [15]. For these reasons, the regulation is seen as a potential remedy for certain issues. However, there are also anticipated barriers to the market introduction of SLB applications. Respondents mentioned that the mandatory use of recycled content (i.e., cobalt, lithium, nickel) in new LIBs can decrease the availability of EoFL LIBs for repurposing as these LIBs are directly transferred to recycling. Similarly, overall recycling rates may also impact the quantity of EoFL LIBs available for repurposing [53].

A specific measure of the new EU Battery Regulation to address prevailing environmental issues is the provision of information on the battery, including on its second-life, in the form of a battery passport by 2026 [15]. However, experts anticipate market entry barriers and expressed concerns about insufficient coordination among relevant stakeholders during the battery passport development, issues related to confidentiality, data liability, accessibility, reliability, and quality. Privacy issues exist for both OEMs and users, who may be hesitant to share sensitive information [54]. In general, experts view the current focus of the battery passport as being more on recycling than on strategies for lifetime extension. This is reflected in the three battery passport pilots launched by the Global Battery Alliance (GBA), which presently only consider static parameters (i.e., already in LIB embedded data at market launch) such as cell type or nominal capacity, but not dynamic parameters (i.e., data generated during use phase) such as a cycle or temperature histogram over a lifetime [55].

#### 3.1.5 Addressing political and regulatory barriers to market entry

To overcome political and legal barriers to a successful market entry of SLB applications, the necessary institutional framework conditions listed in Table 1 could be identified.

**Table 1:** Framework conditions for addressing political and regulatory market entry barriers.

**Institutional framework conditions**	**Content derived from**	
	**Expert interviews**	**Literature**
Promote harmonised policies to support second-life when applicable		[43]
Implement and update testing standards		[54]
Implement standard LIB formats with respective minimum functional and safety requirements		[56]
Introduce design standards for LIB components, including modularity and ability to disassemble as well as format compatibility to ensure interchangeability (e.g., from EVs to BESS)	x	[43]
Implement standards for communication protocols, control architecture, and monitoring		[57]
Implement standardised methodologies for material labelling		[23]
Develop common labels for SLBs as foreseen in the new EU Battery Regulation	x	[15]
Highlight second-life requirements in transport-related regulations, standards, and guidelines		[52]
Encourage decentralised processing of SLBs to reduce problems of dangerous goods transport	x	[53]
Ensure legal compliance of battery manufacturers		[43]
Implement an extended producer responsibility, which is covered to some extent by the new EU Battery Regulation		[[5], [15], [43], [58]]
Implement direct and indirect governmental interventions (e.g., subsidies, taxes)		[[43], [53]]
Introduce mandatory and realistic second-life quotas, either through a total number of SLB applications or a certain proportion of second-life components in new applications	x	
Include clear regulations for permissions of second-life instalments		[43]
Introduce guidelines for the integration of SLB applications in households and industry		[43]
Ensure coordination among all relevant stakeholders during the development of the battery passport and define clear responsibilities	x	
Build a battery passport infrastructure (e.g., data platform, traceability systems)	x	
Introduce marking/ identification rules to provide information on the battery	x	
Mandate disclosure of initial system values and battery history data (e.g., internal resistance increase, capacity, safe operating limit violation) in the battery passport to authorised stakeholders for the determination of SoH, SoS, and RUL	x	
Define clear protocols for the collection of relevant LIB data and ensure high-quality of static and dynamic data	x	
Create a legal framework for battery passport data protection, data transmission, and the application of digital technologies	x	
Ensure transparency, accessibility, and applicability of the battery passport	x	

Standards should be collaboratively developed by regulators, OEMs, research institutes, and repurposing companies [59] and, if possible, on an international level – e.g., coordinated through and published by the Technical Committee (TC) 21 or 21A of the International Electrotechnical Commission (IEC). Moreover, the development of testing standards needs to consider the varying quality of EoL LIBs [54]. However, it is important to avoid overly restrictive design standards at this innovation stage to allow room for new developments, given that LIB production is still undergoing optimisation. The implementation of mandatory second-life quotas remains a subject of controversy. Among the experts consulted, there are differing opinions, with some advocating for this approach while others prefer indirect governmental interventions [43].

### 3.2 Economic market entry barriers

#### 3.2.1 Cost and performance competition with first-life applications

*“Second-life is associated with* significant *manpower. It is less a question of raw materials and energy. It is an economic consideration as well as a question of skilled workers and feasibility, not an ecological one.”* (interviewed ExpK; cf. Table S1 in the Supplementary material)

According to experts and consistent with the literature, one of the most important barriers for second-life manufacturers is price competition with FLB applications [5,8,54]. However, the falling cost curve of FLBs due to optimised production methods and low raw material prices has slowed down since 2020 [60]. Besides battery acquisition costs, SLB manufacturers have to consider additional cost factors. Here, experts consider costs for skilled manual labour required for the disassembly, testing, and reassembly of EoFL components [5,31,61] and the costs associated with expired warranties and product liabilities [25,54] as particularly influential. Currently, the financial risk lies with the installers of second-life systems rather than with battery manufacturers, as is the case with FLBs [43]. These risks necessitate investments in appropriate insurance, but most insurance companies currently do not cover lithium fires. Specific testing and/or modelling of the state of health (SoH) of EoFL cells is necessary when cell manufacturers or OEMs do not provide any information on the battery's history. Other cost factors for repurposing mentioned in interviews include the lack of investors due to multiple crises and the costs for new components. Furthermore, experts noted a scarcity of readily available components for SLB applications, such as balancing systems or battery management systems (BMS), which require substantial development efforts. Concerning supply chains, most available components are currently from Asia rather than Europe. Location also plays a crucial role when it comes to transportation routes of second-life components, as longer distances can potentially increase costs and lead to unnecessary delays [25]. Finally, constantly evolving technology is seen as a barrier to second-life, as a repurposed application must compete in performance with a new application.

#### 3.2.2 Cost competition with recycling and direct reuse

*“What do I get for the raw materials and what do I have to pay for the new battery, the recycling, etc.? The race has not yet been decided, that will become clear in the next few years.”* (interviewed ExpB; cf. Table S1 in the Supplementary material)

The expert opinions on the impact of recycling on the market viability of SLB applications vary and in some cases are even contradictory. In the view of one expert group, a barrier to market entry of SLB applications lies in the limited raw material extraction combined with rapidly growing e-mobility, which results in the preference of recovering secondary raw materials for the production of new LIBs over repurposing. In addition, they see low recycling costs and the financial return from extractable cobalt and nickel as factors that hinder SLB applications and favour recycling options. This is further supported by economies of scale and lobbying efforts leading to substantial investments in new recycling facilities. Economic advantages also result from the lower disposal costs due to recycling, which leads to lower total EV ownership costs [62]. In this context, however, it should be mentioned that the recycling of LIBs is mandatory and must amount at least 65 % of the average weight by December 31, 2025 at the latest [15]. A reduction of the total EV ownership costs may also apply for repurposing [5,20,21], and if both R-strategies are used, the benefits could be cumulative. Further benefits to repurposing are seen by the other expert group, which anticipates that the production capacity of primary LIBs will continue to exceed the available recycling capacity in the forseeable future. This market development would favour the production of SLB applications and delay the recycling of materials for a few years. Present recycling inefficiencies and profitability issues, particularly for certain materials (e.g., lithium, cobalt), may further contribute to this delay, especially when recycling targets cannot be met, resulting in penalties. Considerable research and development efforts are underway to improve and upscale recycling technologies on industrial level [63]. Therefore, experts expect significant progress and a recycling boom within the next decade.

The findings of a recent study emphasised the potential competition between repurposing and direct reuse. With the advancements in battery technology leading to increased ranges, even LIBs with a SoH of 70–80 % retain a significant residual range that can fulfil the requirements of certain customers [54]. This direct use approach offers even greater environmental advantages than repurposing since it has the potential to eliminate the need for any repurposing steps [54]. However, depending on the remaining capacity after one or more direct reuse phases, there may still be sufficient capacity for repurposing.

#### 3.2.3 Business and ownership models

“*OEMs will not let the batteries out of their hands because they are currently investing a lot of money in battery technology and battery plants. OEMs are more likely to operate through subsidiaries with a different name to avoid negative news in the event of a thermal runaway.”* (interviewed ExpG; cf. Table S1 in the Supplementary material)

Martinez-Laserna et al. (2018) identified three LIB ownership models: (i) LIB owned by the EV owner; (ii) LIB leased by the OEM to the EV owner; or (iii) LIB leased by a third party to the EV owner [8]. Experts agreed that the ‘ownership of the OEM’ model gives power to OEMs due to their expert knowledge and battery data ownership [27]. In this respect, most OEMs are reluctant to share not only their knowledge but also their LIBs containing valuable raw materials, as they have invested a lot of money in their technologies. Concerns about insights into patented properties through LIB disassembly and potential reputational damages due to lacking security mechanisms limit OEMs' willingness to engage with external second-life providers. As a result, according to experts, OEMs prefer to explore SLB applications through their own initiatives or subsidiaries. Furthermore, non-disclosure of BMS interface communication is perceived as another barrier [27], although some exceptions exist in collaborations. In any case, a new BMS must be installed, or the software must be reconfigured for the SLB application. Implementing a new BMS is viewed differently among experts, ranging from relatively easy for a BESS to a challenging and costly task. A LIB design that allows for easy separation of the modules would help to equalise the discussion on BMS.

Ownership models are a part of business models, which for their part also influence the marketability of SLB applications. Traditional linear business models prioritise the initial use of batteries rather than their subsequent use, making second-life considerations difficult [10]. According to experts and literature, internal company structures are not conducive to implementing circular business models, with short-term profitability, lack of commitment from top management [64,65], and insufficient communication and coordination within the EV LIB supply chain [31] being major barriers. Furthermore, there is a shortage of trained workers [65] and a lack of a performance metrics system specific to the second-life sector [31].

#### 3.2.4 Availability of end-of-first-life lithium-ion batteries

*“Second-life batteries are not really available on the market yet. If they are, they are purchased from car manufacturers.”* (interviewed ExpC; cf. Table S1 in the Supplementary material)

Experts highlighted that the availability of EoFL LIBs can impede repurposing and SLB market entry. To succeed, SLB applications require a critical amount of similar EoFL LIBs in terms of performance, in particular SoH, state of safety (SoS), and battery chemistry. While combining different LIB chemistries is possible, equal chemistries are preferred to minimise technology costs. Experts believe that due to the prevailing ownership structures (see chapter 3.2.3.), OEMs are currently the main source of organised and single-variety EoFL LIBs. This makes it challenging for external parties to gain access to them. In this regard, complex contracts with OEMs, including specifications for secondary use, are also seen as barriers to entering the SLB market. Besides that, there are already EoFL LIB return actions from OEMs for their production of SLB applications, mainly BESS. Depending on the strategic orientation of OEMs, these EoFL LIBs may not be available for externals in the future. While obtaining similar EoFL LIBs from large fleet owners, such as large companies or leasing providers, appears promising, reliance on OEM warranties may keep these LIBs with the OEM. Procurement through other channels, such as SLB marketplaces like Cling Systems, faces challenges due to their small size and limited development [65]. As the big battery return has not yet started due to the time delay of currently used FLBs, it is also perceived as difficult to target waste management companies for collecting similar-functioning EoFL LIBs and testing them for potential SLB markets. In this context, however, the obligation for proper disposal of EoFL LIBs and the associated contracts between waste management companies and OEMs may pose a greater and perhaps insurmountable obstacle in the view of experts. Finally, the export of EoFL LIBs reduces their availability. Sales of LIBs to other countries are driven partly by the (currently) higher recycling efficiencies and hence higher prices that can be achieved in Asia [66], as well as partly by the potential revenues from the (illegal) export of EoL EVs.

#### 3.2.5 Addressing economic barriers to market entry

Table 2 shows identified institutional framework conditions as well as company internal requirements to overcome economic barriers to a successful market entry of SLB applications.

**Table 2:** Framework conditions and requirements for addressing economic market entry barriers.

**Institutional framework conditions**	**Content derived from**	
	**Expert interviews**	**Literature**
Ensure a level economic and market playing field for first- and second-life operators competing in the same market		[43]
Implement direct and indirect governmental interventions (e.g., subsidies, taxes)		[[43], [53]]
Promote sustainability assessments (e.g., environmental and social LCAs, LCC)	x	[43]
Provide a well-functioning infrastructure and (technical) components for repurposing		[53]
Create a legal framework and collateral for second-life warranty/liability conditions		[[43], [67]]
Promote decentralised processing of SLBs to reduce costs of dangerous goods transport as well as transport-related emissions	x	[53]
**Organisational requirements**	**Expert interviews**	**Literature**
Perform LCC and forecasting	x	[43]
Ensure attractive pricing of SLB applications	x	
Perform customer demonstrations to show the service fulfilment of SLB applications and for educational purposes		[59]
Ensure longevity and safety of the SLB application		[43]
Design LIBs with maximum flexibility, reconfigurability, and modularity to plan LIB repurposing processes in advance		[68]
Observe market leaders and innovative companies and assess future technological, regulatory, and economic developments in cooperations	x	
Gather experience and expand expertise in the second-life area	x	[31]
Ensure a commitment to second-life from top management		[[64], [65]]
Establish new and circular business models for LIBs (e.g., ‘X as a Service’) and for sharing of relevant LIB data (e.g., SoH, SoS)	x	[43]
Develop a performance metrics system for SLB applications		[31]
Offer training for the education of skilled workers (e.g., disassembly, installation)		[31]
Implement collection and take back systems for EoFL LIBs, which is covered by the new EU Battery Regulation	x	[[15], [43]]
Introduce (high-priced) deposit systems for EV LIBs		[53]
Develop and improve marketplaces for EoFL LIBs		[65]
Entering into collaborations with OEMs to leverage existing data and knowledge, access LIBs, and jointly design actions to promote second-life	x	[54]

Sustainability assessments, using life cycle analysis methods for economic, social, and environmental aspects, should be conducted to decide which EoL option is the most sustainable. The involved experts considered it important to provide subsidies for such an assessment, at least in the first few years, as second-life is currently not an established business model. After a few years, stakeholders engaging in the EoL sector would then know which option is more sustainable even without extensive assessments. A purely mandatory approach is perceived as a legal and administrative obstacle [43]. The target cost window of SLB applications should be defined by considering the upper end to be the price of a new LIB, possibly with a discount, and the lower end based on the recycling value. A further discount should be applied to account for safety concerns and the shorter remaining useful life (RUL) of the SLB. A targeted take-back system via local collection systems, producers, or retailers can enable, increase, and simplify collection activities to bundle material flows and finally increase the quantity of available EoFL LIBs for SLB applications [43]. This approach is already being adopted by some OEMs for recycling purposes.

### 3.3 Social market entry barriers

*“Cell phones are collected and taken apart, but nobody wants a second-life cell phone battery. Why should it be any different with second-life car batteries [for energy storage]?”* (interviewed ExpD; cf. Table S1 in the Supplementary material)

Few social barriers to market introduction were identified in the expert interviews. One of these barriers is the public acceptance of SLB applications, which is currently estimated to be rather low. This was attributed to the prevailing attitude of preferring new applications over old ones. According to the literature, a lack of knowledge and communication about the social impacts of SLB applications is considered a contributing factor [10]. Additionally, concerns from potential users about safety and associated risks, for example, caused by thermal runaway or accidents, pose another challenge that needs to be addressed for a successful market launch.

Promotional measures play a pivotal role in facilitating the market entry of SLB applications and encouraging their adoption. In this regard, experts suggest implementing the external and internal measures listed in Table 3.

**Table 3:** Framework conditions and requirements for addressing social market entry barriers.

**Institutional framework conditions**	**Content derived from**	
	**Expert interviews**	**Literature**
Promote SLB applications on a political level for increased public acceptance	x	[43]
Transfer knowledge on SLBs, especially regarding safety, environmental, and social impacts, among different stakeholders from the LIB value network		[[23], [43]]
Improve communication on relevant regulations		[43]
**Organisational requirements**	**Expert interviews**	**Literature**
Ensure fair working conditions in terms of salary, occupational health and safety aspects, working hours, and gender equality	x	[69]

### 3.4 Safety-related market entry barriers

*“If many modules are connected next to each other, thermal propagation is an issue. In a first-life battery, in which the modules are all the same, everything is coordinated, everything has been simulated (e.g., gas propagation, heat conduction). Someone would have to do all this for a second-life application too - perhaps less rigorously if the container is ‘in the field’.”* (interviewed ExpI; cf. Table S1 in the Supplementary material)

There is a lack of experimental data on ageing processes and performance, especially regarding the prediction of battery failure during second-life and reaching the ageing knee point [8]. Such incidents can lead to increased safety risks, including non-functioning, fires, and explosions, during transportation, disassembly and repurposing, the second use phase, as well as EoL [5]. Interviewees expressed concerns about higher residual safety risks in SLBs compared to FLBs, as components in SLBs have aged and key factors influencing safety may have changed (e.g., material degradation, solid electrolyte interphase thickening, lithium plating) [70]. Aged batteries would then require new safety evaluation and simulations such as gas emission, heat transport, or thermal runaway propagation. During the expert interviews, industrial use tended to be considered more promising than private use, as more safety equipment is available. However, a glance at the current market also shows the installation of EV LIBs in home storage systems, mostly coupled with photovoltaic systems. For NMC chemistries in particular, one expert sees no marketability at all, due to their inherent risk potential that can only be controlled (not completely) with great effort. Finally, the level of repurposing (see chapter 3.5.2.) has an impact on safety. It is considered safe to use whole packs or several modules, but not to mix different cells.

These safety-related barriers to market entry can be addressed by implementing the institutional and organisational measures listed in Table 4.

**Table 4:** Framework conditions and requirements for addressing safety-related market entry barriers.

**Institutional framework conditions**	**Content derived from**	
	**Expert interviews**	**Literature**
Define and implement harmonised safety standards for SLB applications	x	[43]
Define and standardise methods and protocols for safety testing of SLBs	x	
Define responsibilities for safety issues along the SLB value cycle		[43]
Develop standardised and well-communicated fire safety measures for SLBs	x	
Develop SLB application-specific guidelines for dealing with thermal propagation	x	[43]
Develop mitigation strategies for cell-to-cell propagation in SLB applications		[43]
Develop guidelines for catastrophic events like flooding situations		[43]
Train authorities and installation workers for second-life instalments on industrial and household level		[43]
Offer dedicated training sessions for first responders regarding propagation, outgassing, and high-voltage issues	x	
**Organisational requirements**	**Expert interviews**	**Literature**
Increase experimental tests to gather data on ageing performance		[8]
Perform safety assessments on SLBs to ensure the same level of safety as for FLBs	x	[43]
Incorporate safety functions into SLB applications (e.g., shutdown of certain cells)	x	
Integrate an early safety warning detection system in SLB applications, possibly in conjunction with an extinguishing system		[43]
Install outgassing control mechanisms for emergencies, especially to avoid toxic gases and the associated negative environmental impacts		[43]
Define a safe operating temperature range for SLB applications		[43]

Ageing experiments are crucial for developing ownership and business models, determining appropriate warranty periods, and making lifetime predictions [8]. Moreover, experts suggested that distinguishing between different LIB chemistries as well as business-to-business (B2B) and business-to-consumer (B2C) would be beneficial in addressing safety concerns. In any case, ensuring the safety of SLB applications is considered imperative. By allowing only safe SLB systems that adhere to relevant standards and regulations to enter the market, the overall acceptance of SLB applications in society is expected to increase.

### 3.5 Technical market entry barriers

#### 3.5.1 Complexity of battery design and requirements

*“Not every battery is identical: different voltage levels, capacities, cell chemistries, etc. This needs to be considered by regulations to ensure safety. There is a need for basic requirements.”* (interviewed ExpB; cf. Table S1 in the Supplementary material)

Battery designs are manufacturer- and application-specific, resulting in complexity. According to experts, mixing different cell chemistries, specifically the currently most frequently used NMC and LFP cells, is difficult and sometimes even impossible due to their differing voltage levels. Variations in the cells are also present due to different ageing histories. This further raises a question of BMS liability should the different cells be incorrectly controlled and, in the worst case, a thermal runaway occurs. Additionally, the variety of system architectures but also gap fillers, potting compounds, and welded components currently hinder the automated disassembly of EoFL LIB components in the view of experts. The continuous advancement of LIB technologies and changing designs contribute to the heterogeneity of EoFL LIBs, which inhibits interchangeability and increases complexity and costs for repurposing companies. Individual solutions to design complexity are currently deemed economically infeasible by experts. Moreover, according to the literature, the ongoing progress in EV LIBs creates discrepancies in meeting the demands of both FLB and SLB applications. EV LIBs are increasing in size and undergoing optimisation to enhance energy density rather than their cyclic stability. However, in SLB applications, particularly for BESS, maintaining a high level of cyclic stability is crucial [54]. In this context, Börner et al. (2022) suggest that EoFL LIBs from long and medium-range electric trucks are more promising for a second use (i.e., BESS) than from electric passenger cars due to their similar operating conditions and good SoH of around 70–80 % [54].

#### 3.5.2 Level of repurposing

*“Second-life becomes more difficult when integration is no longer cell-to-module but directly cell-to-pack. This is the worst case for a second-life as the battery can then only be used as a whole. It cannot be dismantled without destroying it. In this case, the primary application comes first.”* (interviewed ExpI; cf. Table S1 in the Supplementary material)

Disassembly can be done at different levels: cell, module, and pack. Using complete packs for a second use requires less effort and lower costs, but there is a risk of performance loss due to weaker subordinate elements. If disassembly goes deeper into modules or cells, more effort and additional components are needed for repurposing activities. Moreover, it increases safety risks during use as well as the risk to workers involved in repurposing. However, according to experts, this allows for sorting out weaker elements or reassembling LIBs based on capacity. Currently, most players involved in repurposing use modules for their SLB applications due to economic feasibility, maintenance of certifications, safety issues, and technical complexities. The shift towards cell-to-pack or cell-to-chassis architectures in LIBs reduces the possibility of non-destructive disassembly, mainly limiting it to the pack level. This is seen as conflicting with current regulatory efforts focused on circular economy principles. One interviewee considered this shift a ‘worst-case scenario’ for SLB applications as a non-destructive disassembly will be hardly possible with such system architectures. Since modularity is an important design criterion for a second use, but only subordinate to recycling, experts assume that these trends will favour recycling over repurposing. The impact of this trend is expected to be strongest in the automotive sector, particularly for high-volume vehicle series.

#### 3.5.3 Evaluation of end-of-first-life lithium-ion batteries

*“For a reliable assessment, you need the initial condition and life log of the battery - OEMs have the data (hidden in some source code) but do not pass it on.”* (interviewed ExpD; cf. Table S1 in the Supplementary material)

Assessing the suitability of EoFL LIBs for SLB applications requires evaluating parameters like SoH, SoS, and RUL. According to experts and literature, however, determining the RUL is complex due to variations in use cases [5,8]. The lack of a universally valid definition for SoH and SoS, as well as the limited access to initial system values and dynamic data from the use phase further complicate the assessment. Some companies argue that OEM/manufacturer data is unnecessary, although this claim is controversial. In particular, scepticism was expressed about the accurate calculation of the ageing knee point without OEM data, which is considered difficult even with OEM data. However, in the absence of battery history data, companies in the second-life market have developed their own test methods for second-life suitability.

#### 3.5.4 Addressing technical barriers to market entry

To overcome technical barriers to a successful market entry of SLB applications, the institutional framework conditions and organisational requirements listed in Table 5 could be identified. Measures to address design-related barriers to market entry at an institutional level as well as framework conditions for providing data for an evaluation of an EoFL LIB are presented in chapter 3.1.5.

**Table 5:** Framework conditions and requirements for addressing technical market entry barriers.

**Institutional framework conditions**	**Content derived from**	
	**Expert interviews**	**Literature**
Define the parameters SoH and SoS	x	
Develop an integrated monitoring system for static and dynamic parameters of LIBs		[43]
Develop standardised test methods for the determination of the SoH, SoS, and RUL (preferably in cycles)	x	[43]
**Organisational requirements**	**Expert interviews**	**Literature**
Use modular multilevel converter (MMC) technologies to allow for the combination of LIBs with different cell chemistries, from different production batches, and with different SoH levels	x	
Apply MBSE approaches to optimise the FLB application including a subsequent second-life		[54]
Implement balancing strategies to control different cells		[54]
Implement and/or redesign control and management systems for SLB applications		[[8], [43]]
Reconfigure key control parameters (e.g., voltage limits, current derating)		[54]
Develop an application requirement and technology profile matrix for SLB applications	x	
Ensure an appropriate battery system design for SLB applications		[43]
Group SLBs with similar characteristics such as chemistry, capacity, or internal resistance for the production of SLB applications		[[67], [71]]
Ensure high cyclic stability for SLB applications		[43]
Use advanced digital technologies to model/predict the SoH, SoS, and RUL	x	

An integrated development of multi-life LIB systems using model-based systems engineering (MBSE) approaches involves the integration of thermal, mechanical, and electrical domains to cope with different dependencies between models, as well as between models and requirements [54]. Furthermore, due to the heterogeneities of SLBs as well as increased internal resistance, appropriate management systems need to be considered, such as thermal management systems (TMS) or energy management systems (EMS) [43]. Regarding the use of advanced digital technologies, such as blockchain technologies for tracking the LIB flow history or machine learning for intelligent data management, data security and protection issues must be addressed [67].

## 4 Conclusions

This study adopted an unprecedented method within the field of SLBs, merging expert and problem-centred interviews with an explorative workshop. This unique approach facilitated the acquisition and revelation of first-hand insights regarding barriers, necessary institutional framework conditions, and organisational requirements for repurposing. As deduced from the study, a second-life does not conflict with subsequent recycling but is an important element of the circular economy and should be preferred according to the waste hierarchy as outlined in the Waste Framework Directive, with reuse preceding recycling and disposal. A functioning circular economy involves all parties along the value cycle and includes various strategies to extend the lifespan of products and materials, maximising their value. As there are many external and internal influencing factors, a comprehensive transition to a circular economy using a one-size-fits-all approach is impossible. In the specific case of repurposing, many of these influencing factors are ultimately translated into costs. Therefore, despite their many environmental benefits, the profitability and scalability of SLB applications currently face challenges. According to several experts, it will be an economic trade-off whether or not to repurpose EoFL LIBs, particularly in the absence of regulatory incentives. Consequently, it is crucial to establish a broad selection of supportive framework conditions and policy actions that promote the adoption of second-life concepts and emphasise the role of the circular economy. Furthermore, new emerging battery or recycling technologies may alter the evaluation of the market situation in the future. Besides these external influencing factors, it is important that life extension strategies should be considered during the design phase, such as ‘design for disassembly’, ‘design for repurposing’, or ‘safe and sustainable by design’. In this respect, further in-depth studies are needed at the scientific level, focusing on design-related strategies. Moreover, other strategies for lifetime extension should be analysed regarding their barriers to market entry including research on adequate key performance indicators (KPIs) to assess the successful market launch of SLBs and their applications. Additionally, it would be interesting to analyse the job creation potential through an increased application of second-life concepts. Lastly, on a technical level, an overall accepted definition of SoH and SoS as well as gathering more data on ageing mechanisms is essential for facilitating repurposing efforts and ensuring the safety of SLB applications. Overall, the findings provide guidance for diverse stakeholders involved in the repurposing sector. Policymakers and regulators can utilise the proposed measures at an institutional level to establish the necessary framework conditions for achieving a genuine circular economy. Within companies, the study offers potential solutions for overcoming existing market entry barriers at an organisational level. Although the focus of this study was on European stakeholders, most of the general results seem to be applicable globally. Specific results can be directly extrapolated to other regions with similar socio-economic and regulatory conditions.

## CRediT authorship contribution statement

**Stefanie Prenner:** Writing – original draft, Visualization, Methodology, Investigation, Formal analysis, Data curation, Conceptualization. **Florian Part:** Writing – review & editing, Validation, Supervision, Project administration, Methodology, Funding acquisition, Conceptualization. **Sabine Jung-Waclik:** Writing – review & editing, Validation, Supervision, Methodology, Conceptualization. **Arnaud Bordes:** Writing – review & editing, Visualization. **Robert Leonhardt:** Writing – review & editing, Validation. **Aleksander Jandric:** Writing – review & editing, Validation. **Anita Schmidt:** Writing – review & editing, Validation. **Marion Huber-Humer:** Writing – review & editing, Validation, Supervision.

## Declaration of competing interest

The authors declare that they have no known competing financial interests or personal relationships that could have appeared to influence the work reported in this paper.

## Figures and Tables

**Table:** 

B2B	Business-to-business
B2C	Business-to-consumer
BESS	Battery energy storage system
BMS	Battery management system
EMS	Energy management system
EoFL	End-of-first-life
EoL	End-of-life
EV	Electric vehicle
FLB	First-life lithium-ion battery
GBA	Global Battery Alliance
IEA	International Energy Agency
IEC	International Electrotechnical Commission
KPI	Key performance indicator
LCA	Life cycle assessment
LCC	Life cycle costing
LFP	Lithium iron phosphate
LIB	Lithium-ion battery
MBSE	Model-based systems engineering
MMC	Modular multilevel converter
NMC	Lithium nickel manganese cobalt oxide
OEM	Original equipment manufacturer
PESSTEL	Political, economic, social, safety-related, technological, environmental, and legal factors
PESTEL	Political, economic, social, technological, environmental, and legal factors
PEV	Plug-in electric vehicles
RUL	Remaining useful life
SDG	Sustainable Development Goal
SLB	Second-life lithium-ion battery
SoH	State of health
SoS	State of safety
SPIN	Situation-problem-implication-need
TC	Technical committee
TMS	Thermal management system
UN	United Nations
UNDP	United Nations Development Programme
