# Supplementary material for: Barriers and framework conditions for the market entry of second-life lithium-ion batteries from electric vehicles
Source: Heliyon. 2024 Sep 7;10(18):e37423. doi: 10.1016/j.heliyon.2024.e37423 (PMC11416477; doi:10.1016/j.heliyon.2024.e37423)
Supplement: Supplementary file 1 [file mmc1.pdf]

Barriers and framework conditions for the market entry of second-life lithium-ion batteries from electric vehicles

Supplementary material

Stefanie Prenner [1], Florian Part *, [2], Sabine Jung-Waclik [1], Arnaud Bordes [3], Robert Leonhardt [4], Aleksander Jandric [2], Anita Schmidt [4], Marion Huber-Humer [2]

[1] Brimatech Services GmbH, Lothringerstraße 14/3, 1030 Vienna, Austria
[2] BOKU University, Institute of Waste Management and Circularity, Muthgasse 107, 1190 Vienna, Austria
[3] Institut National de l'Environnement Industriel et des Risques (Ineris), Parc Technologique Alata, BP2, 60550 Verneuil-en-Halatte, France
[4] Bundesanstalt für Materialforschung und -prüfung (BAM), Unter den Eichen 87, 12205 Berlin, Germany

** Corresponding author: Florian Part, florian.part@boku.ac.at*

1. Data collection

1.1. Literature research

Extensive literature research was conducted on the following topics and keywords:

- Stakeholders of (second-life) lithium-ion battery applications
- Value networks / value chains / value cycles of (second-life) lithium-ion batteries
- Drivers and barriers for a successful market introduction of second-life lithium-ion battery applications
- Organisational requirements and prerequisites as well as institutional framework conditions for a successful market launch of second-life lithium-ion battery applications
- Regulation, standards, and strategies for (second-life) lithium-ion batteries

To discover pertinent scientific articles, along with other relevant documents and information, a systematic literature search was conducted using the following methods: (i) searching scientific online databases (e.g., Scopus, Google Scholar), (ii) directly exploring subject-specific journal websites (e.g., Energy, Batteries, Heliyon, Journal of Applied Energy), (iii) investigating project websites related to second-life lithium-ion batteries (SLBs) (e.g., SASLAB, Lions2Life), (iv) exploring websites of relevant clusters, associations, initiatives, and platforms (e.g., Batteries European Partnership Association [BEPA], Global Battery Alliance [GBA]), (v) directly visiting original equipment manufacturers' (OEMs) websites (e.g., BMW, Renault), (vi) exploring standardisation websites (e.g., Underwriters Laboratories [UL], International Organization for Standardization [ISO]), and (vii) visiting event and conference websites related to SLB topics (e.g., Battery Tech, International Congress for Battery Recycling). During the literature research process, the keywords outlined in the aforementioned list were utilised and combined in various ways. Following an initial systematic search, an additional research step was performed, employing the snowball principle, whereby previously discovered documents were used to identify further sources of relevant information and documents.

1.2. Qualitative interviews

1.2.1. Number and selection of interview partners

Griffin and Hauser (1993) suggest that approximately 15 one-hour, face-to-face interviews cover about 80 % of the relevant aspects, while 20-30 interviews are needed for about 90-95% coverage [1]. Alternatively, Zaltman and Higie (1993) recommend 7-15 one-to-one interviews lasting 90-120 minutes [2]. However, the necessary number of interviews varies based on specific research questions. This study followed the principle of theoretical saturation, meaning that no significant additional knowledge was gained from further interviews. This implies that the interviews did not reveal any new properties or dimensions related to the specific topics and categories of the interview guide (see chapter 1.2.3) and that the interviews accounted for much of the possible variability [3]. Theoretical saturation in this study was achieved after 13 interviews, which all were conducted anonymously between January and December 2022. Further details on the interviewees' profiles can be found in Table S1.

Table S1: The interviewees' profile (n=13). Organisation size: micro (up to 9 employees), small (10-49 employees), medium (50-249 employees), large (250-499 employees), very large (500 employees and more).

Expert ID	Expertise in the LIB value network	Organisation type	Organisation size	Duration
ExpA	Supply first-life application	Company	Medium	60 min
ExpB, ExpC*<br>*Simultaneous interview with 2 experts	Production of FLBs	Company	Medium	60 min
ExpD	Production of FLBs; Repurposing of EoFL LIBs	Company	Medium	80 min
ExpE	Energy supply	Company	Very large	70 min
ExpF	Research & development	Research institute	Very large	60 min
ExpG	Research & development; Standardisation & regulation	Research institute	Very large	90 min
ExpH	Consultancy in the field of batteries	Company	Very large	80 min
ExpI	Modular power electronics	Company	Small	60 min
ExpJ	Research & development; LIB testing & analysing	Company	Very large	70 min
ExpK	Repurposing of EoFL LIBs	Company	Medium	60 min
ExpL	Repurposing of EoFL LIBs	Company	Small	60 min
ExpM	Recycling and pre-treatment of EoL LIBs	Company	Very large	60 min

Table S1 shows the interviewees' wide range of different perspectives, in particular supply and production of first-life lithium-ion battery (FLB) applications, repurposing of end-of-first-life (EoFL) lithium-ion batteries (LIBs), recycling, research and development (R&D), regulation and standardisation, energy supply, modular power electronics, LIB testing and analysing, and consultancy. By adopting this approach, the data quality was enhanced and diverse insights were obtained. The validity of data collection was maintained through rigorous analysis of the interaction process, including examination of indicators of authentic or non-authentic behaviour within the interview recordings. In addition, the validity of the data was further enhanced by using multiple independent recorders/observers for the same behavioural sequence as well as additional interviews [4].

1.2.2. Expert interviews and problem-centred interviews

Expert interviews are an effective method for collecting qualitative data in empirical social research, especially when investigating the knowledge of experts responsible for specific problem solutions [5,6]. This method was chosen for the present work because it is frequently used in industrial sociological research [6] and offers the possibility of gaining exclusive insider insights into structural connections and processes of change within systems of action [7].

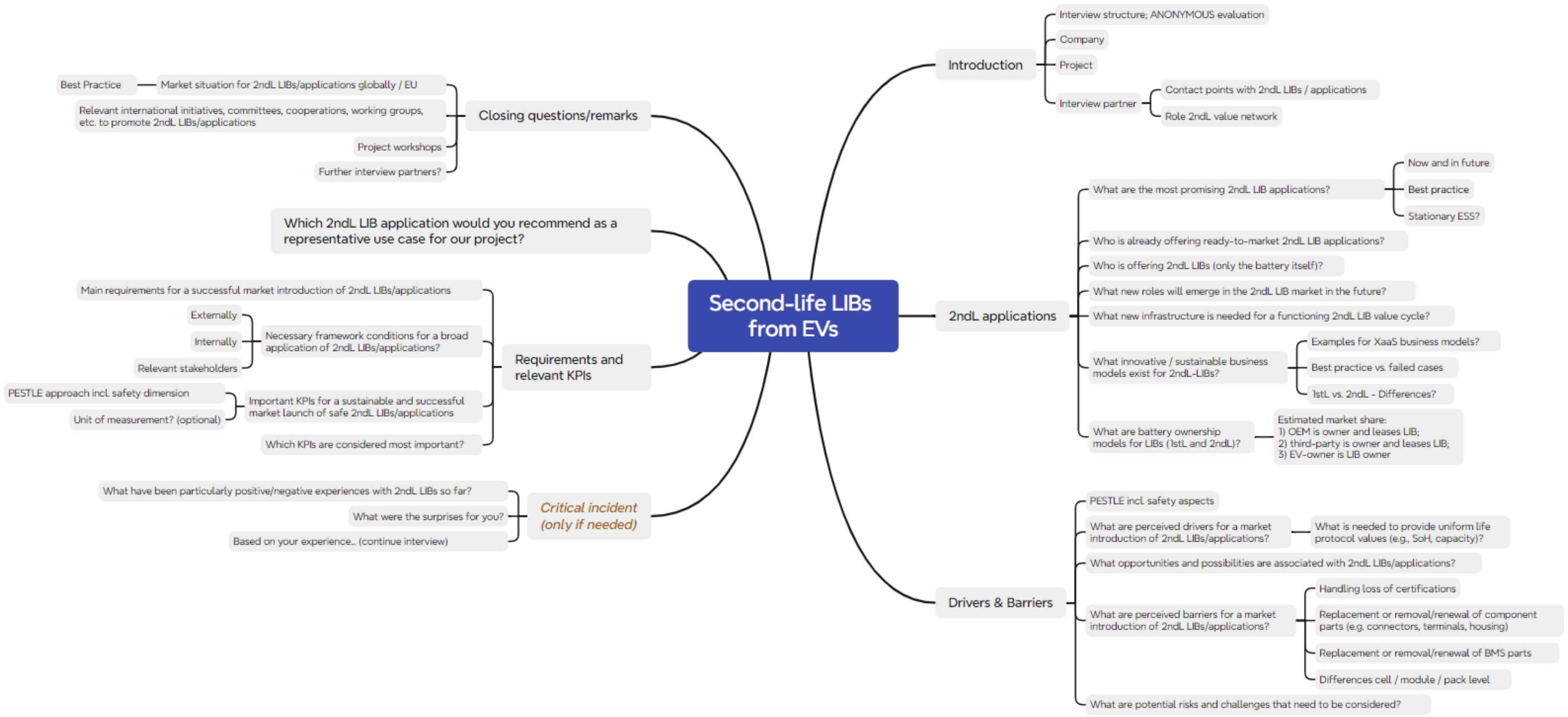

Figure S1: Interview guide for expert and problem-centred interviews.

The problem-centred interview technique, introduced by Witzel (1982), aims to gather a comprehensive and thorough collection of information related to a predefined problem area [8,9]. This method is particularly useful for a theory-based approach and for addressing questions within a poorly defined object area, rather than purely exploratory ones. By employing the problem-centred interview, it becomes possible to identify structures, trends, and conditions within these areas [8]. The significance of this technique for the current study arises from its frequent utilisation in addressing issues within the high-tech sector [8].

1.2.3. Structuring methods and approaches

Qualitative interviews can be structured using different techniques. An interview guide is an essential element for maintaining structure and situational relevance [9,10]. It serves as a thematic outline of the entire problem area, acting as a memory aid and providing orientation throughout the interviews. The guide ensures comparability and control across different interviews [9]. Visualising the interview guide using a 'mind map' is a common practice [8]. Additionally, the guide proves useful when conversations with interview partners become stagnant or veer off course [9]. Figure S1 shows the interview guide developed and used for this study.

1.3. Project meetings

Project internal experts from the research team actively engaged and participated in bi-monthly meetings. These experts possess specialised knowledge, skills, and experience in the field of (second-life) LIBs, which aligns with the objectives and requirements of this work. Involving these experts allowed for leveraging their existing expertise to discuss, validate, and enhance previous knowledge and research findings.

2. Data evaluation and analysis

Qualitative interviews and explorative online workshops generate transcripts, which can be analysed through qualitative content analysis. Content analysis is an empirical approach to analysing texts in their communication context. It follows rules and step-by-step models, emphasising interpretation without hasty quantification [11]. Kuckartz (2010) outlines the essential elements of scientifically evaluating qualitative data as follows: exploration, interpretation, categorisation, classification (including type formation), and theory construction [12]. In combined expert and problem-centred interviews, knowledge is gained through an inductive-deductive relationship in both the elicitation and evaluation processes. Despite the absence of an evaluation method specifically designed for these survey methods, there are various analysis techniques available that cater to different epistemological interests [13]. There is also no specifically recommended evaluation methodology for the data collected in workshops. For this study, qualitative content analysis following Mayring (2000) was chosen [11].

Mayring's framework distinguishes between two types of category development: inductive and deductive [11]. The step models for both approaches are illustrated in Figure S2. Both approaches, inductive and deductive category development, were applied during the content analysis. In the inductive method, categories were directly derived from the research material without any reference to pre-existing theoretical concepts. On the other hand, the deductive approach involved forming and defining categories before analysing the data. These categories were based on pre-established, theoretically derived aspects of analysis, which were then applied to the text [11].

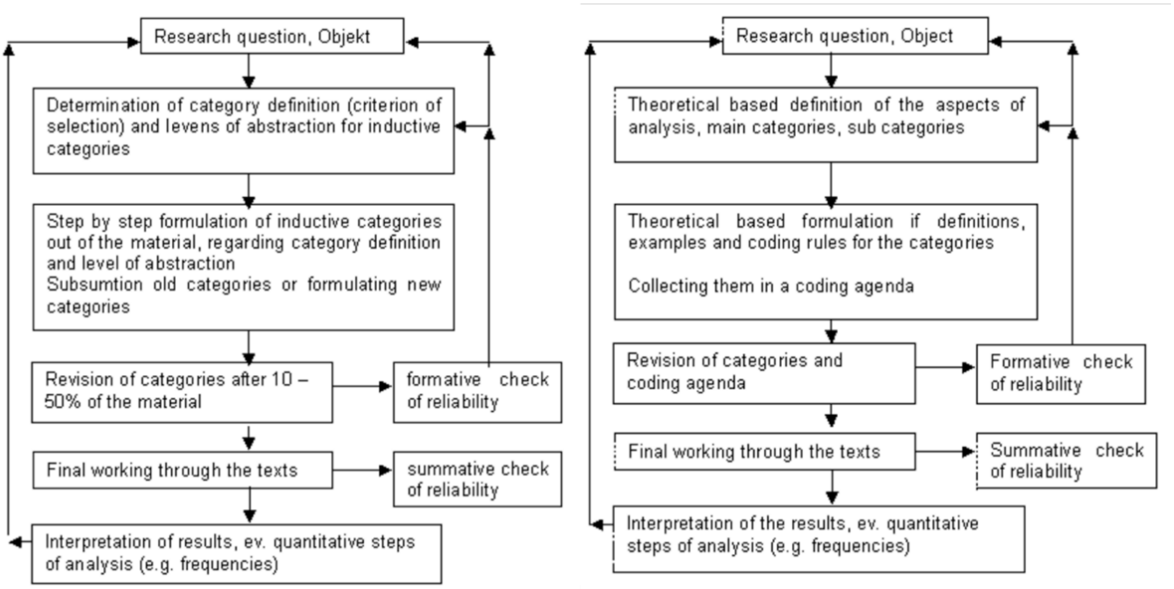

Figure S2: Step models of inductive (left) and deductive (right) category application [11].

Moreover, throughout the analysis process, these categories were open to expansion, supplementation, or even new development. The continuous feedback loops facilitated ongoing revisions of the categories, enabling the creation of overarching categories that encompassed broader themes and connections within the data. This iterative approach ensured that the analysis remained dynamic and comprehensive, capturing the richness and complexity of the research material. A total of 45 categories were formed, which relate to 7 category groups and are shown in Table S2. For coding the transcripts based on the developed categories the software tool ATLAS.ti version 8.0.27.0 was used.

Table S2: Inductively and deductively developed categories and category groups of the qualitative content analysis.

Categories			
Nr.	***Name***	***Grounded***	***Group***
**1**	(New) battery regulation	7	vi
**2**	First-life application	6	i
**3**	Second-life application future	13	i
**4**	Second-life application now	17	i
**5**	Second-life provider	11	iii
**6**	Third-life battery	3	v
**7**	Battery passport	12	vi
**8**	Battery system	6	v
**9**	b-cells	2	v
**10**	Best practice	12	i
**11**	BMS	19	v
**12**	Business models	35	iii
**13**	Costs	22	iii
**14**	Customer demand	3	iii
**15**	Digital technologies	11	v
**16**	Ecological barrier	0	ii
**17**	Ecological driver	16	iv
**18**	Economic barrier	66	ii
**19**	Economic driver	23	iv
**20**	EoL LIBs	23	iii
**21**	Infrastructure	1	iii; vii
**22**	International initiatives	3	iii
**23**	LIB chemistry & form	15	v
**24**	Marketplace & platform	5	iii
**25**	MMC technology	12	v
**26**	New roles	10	iii
**27**	Ownership models	5	iii
**28**	Political barrier	1	ii
**29**	Political driver	8	iv
**30**	Product interviewee	17	i
**31**	R&D second-life	13	v
**32**	Recycling	23	v
**33**	Regulatory barrier	34	ii
**34**	Regulatory driver	18	iv
**35**	Organisational & institutional requirements	49	vii
**36**	Safety barrier	8	ii
**37**	Safety driver	4	iv
**38**	Social barrier	3	ii
**39**	Social driver	3	iv
**40**	Standards, certifications, tests	26	vi
**41**	Technical barrier	40	ii
**42**	Technical driver	25	iv
**43**	Thermal runaway	9	v
**44**	Trends	20	v
**45**	Use case	5	i

Category Groups		
Nr.	***Name***	***Number of attributed codes***
i	Applications	6
ii	Barriers	7
iii	Business modelling	10
iv	Drivers	7
v	LIBs	11
vi	Regulation	3
vii	Requirements & framework conditions	2

3. Limitations

A few limitations should be acknowledged in the present study. Firstly, the examination of product lifetime extension strategies was confined to the analysis of repurposing exclusively, neglecting the exploration of other R-strategies such as rethink, reduce, reuse, repair, refurbish, remanufacture, recycle, or recover. Additionally, the geographic scope of the study is largely limited to European countries within a global context. Furthermore, it is crucial to note that the literature analysis conducted in this study does not constitute a comprehensive review of all available literature on the topic. Instead, it represents a targeted exploration, focusing on specific dimensions relevant to the study's objectives.

Glossary

BEPA	Batteries European Partnership Association
EoFL	End-of-first-life
EoL	End-of-life
FLB	First-life lithium-ion battery
GBA	Global Battery Alliance
ISO	International Organization for Standardization
LIB	Lithium-ion battery
MMC	Modular multilevel converter
OEM	Original equipment manufacturer
R&D	Research and development
SLB	Second-life lithium-ion battery
UL	Underwriters Laboratories

References

[1] A. Griffin, J.R. Hauser, The Voice of the Customer, Marketing Science 12 (1993) 1–27.

[2] G. Zaltman, R. Higie, Seeing the voice of the customer: The Metaphor Elicitation Technique. Working Paper, Cambridge, MA, Marketing Science Institute (1993) 93–114.

[3] J.M. Corbin, A.L. Strauss, Basics of qualitative research: Techniques and procedures for developing grounded theory, Fourth edition, SAGE, Los Angeles, 2015.

[4] J. Bortz, N. Döring, Forschungsmethoden und Evaluation in den Sozial- und Humanwissenschaften: Für Human- und Sozialwissenschaftler; mit 87 Tabellen, fifth., vollst. überarb. aktual. und erweitere Aufl., Springer, Heidelberg, 2016.

[5] M. Pfadenhauer, Das Experteninterview, in: H. Holzmüller, R. Buber (Eds.), Qualitative Marktforschung: Konzepte-Methoden-Analysen, second ed., Gabler Verlag | Springer Fachmedien, Wiesbaden, 2009, pp. 449–461.

[6] M. Meuser, U. Nagel, Das Experteninterview - konzeptionelle Grundlagen und methodische Anlage, in: S. Pickel, D. Jahn, H.-J. Lauth, G. Pickel (Eds.), Methoden der vergleichenden Politik- und Sozialwissenschaft, VS Verlag für Sozialwissenschaften, Wiesbaden, 2009, pp. 465–479.

[7] R. Liebold, R. Trinczek, Experteninterview, in: S. Kühl, P. Strodtholz, A. Taffertshofer (Eds.), Handbuch Methoden der Organisationsforschung, VS Verlag für Sozialwissenschaften, Wiesbaden, 2009, pp. 32–56.

[8] A. Kurz, C. Stockhammer, S. Fuchs, D. Meinhard, Das problemzentrierte Interview, in: H. Holzmüller, R. Buber (Eds.), Qualitative Marktforschung: Konzepte-Methoden-Analysen, second ed., Gabler Verlag | Springer Fachmedien, Wiesbaden, 2009, pp. 463–475.

[9] A. Witzel, Verfahren der qualitativen Sozialforschung: Überblick und Alternativen. Zugl.: Bremen, Univ., Diss., 1980, Campus Verlag, Frankfurt/Main, 1982.

[10] M. Meuser, U. Nagel, ExpertInneninterviews - vielfach erprobt, wenig bedacht, in: D. Garz, K. Kraimer (Eds.), Qualitativ-empirische Sozialforschung: Konzepte, Methoden, Analysen, Westdeutscher Verlag, Opladen, 1991, pp. 441–471.

[11] P. Mayring, 2000. Qualitative Content Analysis. Forum: Qualitative Sozialforschung / Forum: Qualitative Social Research 1, 20. https://doi.org/10.17169/fqs-1.2.1089.

[12] U. Kuckartz, Einführung in die computergestützte Analyse qualitativer Daten, third., aktualisierte Auflage, VS Verlag für Sozialwissenschaften / GWV Fachverlage GmbH, Wiesbaden, Wiesbaden, 2010.

[13] A. Witzel, 2000. The Problem-centered Interview. Forum: Qualitative Sozialforschung / Forum: Qualitative Social Research 1, 22. https://doi.org/10.17169/fqs-1.1.1132.
